# Calorimetric Study of *Helix aspersa* Maxima Hemocyanin Isoforms

**DOI:** 10.1155/2018/8450792

**Published:** 2018-03-04

**Authors:** Svetla Todinova, Yuliana Raynova, Krassimira Idakieva

**Affiliations:** ^1^Institute of Biophysics and Biomedical Engineering, Bulgarian Academy of Sciences, Bl. 21, Acad. G. Bonchev Str., Sofia 1113, Bulgaria; ^2^Institute of Organic Chemistry with Centre of Phytochemistry, Bulgarian Academy of Sciences, Bl. 9, Acad. G. Bonchev Str., Sofia 1113, Bulgaria

## Abstract

The thermal unfolding of hemocyanin isoforms, *β*-HaH and *α*_*D*+*N*_-HaH, isolated from the hemolymph of garden snails *Helix aspersa* maxima, was studied by means of differential scanning calorimetry (DSC). One transition, with an apparent transition temperature (*T*_*m*_) at 79.88°C, was detected in the thermogram of *β*-HaH in 20 mM HEPES buffer, containing 0.1 M NaCl, 5 mM CaCl_2,_ and 5 mM MgCl_2_, pH 7.0, at scan rate of 1.0°C min^−1^. By means of successive annealing procedure, two individual transitions were identified in the thermogram of *α*_*D*+*N*_-HaH. Denaturation of both hemocyanins was found to be an irreversible process. The scan-rate dependence of the calorimetric profiles indicated that the thermal unfolding of investigated hemocyanins was kinetically controlled. The thermal denaturation of the isoforms *β*-HaH and *α*_*D*+*N*_-HaH was described by the two-state irreversible model, and parameters of the Arrhenius equation were calculated.

## 1. Introduction

Many natural proteins act as a functional basis of biologic drugs [1]. One of the first steps, required to outline the design of the therapeutic properties of protein drugs, is their thermodynamic characterization [2]. The attainment of desired thermodynamic features of proteins is a relatively new area in biotechnology. Thus, protein stabilization becomes a main goal in medicine and scientific research. The mechanism of folding and unfolding gives knowledge on the function and the possibilities of protein stabilization. Differential scanning calorimetry (DSC) is the most useful technique for analyzing the protein thermal stability. This method measures the heat capacity change, associated with protein thermal denaturation, as a function of temperature. The integral of the excess heat capacity function is the enthalpy in this process. Thermodynamic parameters, such as temperature of unfolding (*T*_*m*_) and change in enthalpy, give huge information about the structure and functional parameters of the proteins, as well as about the nature of their interactions with other ligands or drug [3–5].

Hemocyanins (Hcs) are oligomeric copper-containing glycoproteins that function as oxygen carriers in the hemolymph of several molluscs and arthropods [6, 7]. Besides their important biological function, a variety of medical applications of molluscan Hcs emerged. Recently, we have established that the Hcs of marine mollusk *Rapana thomasiana* (RtH) and *β*-Hc isoform of *H. pomatia* (*β*-HpH) are suitable as potential bioadjuvants for subunit vaccines and that these Hcs could be used as natural adjuvants or protein carriers [8, 9]. The anticancer properties of both molluscan Hcs have been demonstrated on a murine model of colorectal cancer *in vivo* [10]. A number of DSC studies have shown that Hcs possess considerable thermal stability. Melting temperature (*T*_*m*_) in the range 83–90°C was observed for the RtH [11], *β*-HpH [12], and the Hc of marine gastropod *Concholepas concholepas* (CCH) [13]. Calorimetric studies demonstrate also the high thermostability of Hcs from arthropods, for example, Hcs of tarantula *Eurypelma californicum* (*T*_*m*_ 90°C) [14] and of horseshoe crab *Limulus polyphemus* (*T*_*m*_ 92°C) [15]. It is considered that the interactions between subunits and the high degree of oligomerization stabilize the quaternary structure of the Hc molecules [15, 16].

In certain species of mollusc, an expression of functionally distinct Hc isoforms has been observed [17]. The physiological relevance of these isoforms is still not clear, but its expression may be connected to the development of species [18]. Heterogeneity at a molecular level has been described first for the Hc from *Helix* species [19]. Three types of Hc molecules or isoforms have been identified in the hemolymph isolated from the Roman snail *Helix pomatia*: *β*-Hc, *α*_*D*_-Hc (dissociating *α*-Hc), and *α*_*N*_-Hc (nondissociating *α*-Hc) [6, 20]. The isoform *β*-HpH consists of only one type of polypeptide chain (*β* subunits) compared with two types (*α* and *α*′ subunits) in each of both *α*-Hcs [20]. Due to the subunit homogeneity, structural and immunological investigations have been performed primarily on *β*-HpH. Gielens et al. have identified three types of Hc components in the hemolymph of garden snails *Helix aspersa* [21]. Three isoforms (*β*-HlH, *α*_*N*_-HlH, and *α*_*D*_-HlH) have been reported also for the garden snail *Helix lucorum* [22]. Recently, the Hc of snails *Helix aspersa* maxima (HaH) was isolated and characterized [23, 24]. On the basis of the o-diphenoloxidase activity of HaH, a model of biosensor for quantitative determination of phenols in aqueous solutions was developed [23]. Moreover, this Hc can be produced in large quantities for biotechnology and medical use from snails, bred in special farms under controlled conditions; therefore, the knowledge of its thermal stability is essential.

In the present study, for the first time, the thermal stability of the individual *H. aspersa* maxima Hc isoforms has been characterized by means of DSC. The data are compared with those obtained for other molluscan Hcs in order to acquire further insight into the structural stability of the oxygen-transport proteins from invertebrates.

## 2. Materials and Methods

### 2.1. Hemocyanin Preparation

Native HaH was isolated in compliance with the procedure described in [23]. Briefly, the Hc was obtained from the hemolymph, collected from snails *Helix aspersa* maxima, by ultracentrifugation at 180000 ×g (ultracentrifuge Beckman LM-80, rotor Ti 45), for 4 hours, at 4°C. The pellets were resuspended in 50 mM phosphate buffer, pH 7.2, and HaH was further purified by gel filtration chromatography on a Sepharose 4B column (90 × 2.4 cm). Further, HaH was separated into its isoforms (*β*-HaH and *α*_*D*+*N*_-HaH) by ion exchange chromatography on a DEAE-Sepharose CL-6B column (32 × 1.2 cm) and equilibrated and eluted with buffer 50 mM Tris-HCl, pH 8.0, using a linear gradient 0.1–0.45 M NaCl. Specific absorption coefficient *a*_278_ nm = 1.413 ml·mg^−1^·cm^−1^ for HaH [21] was used for determination of the protein concentration.

### 2.2. Differential Scanning Calorimetry

Calorimetric measurements were performed using high-sensitivity differential adiabatic scanning microcalorimeter DASM-4 (Biopribor, Pushchino, Russia), with a sensitivity >0.017 mJ·K^−1^and a noise level < ±0.05 *μ*W. Four different scan rates (0.2, 0.5, 1.0, and 1.5°C min^−1^) were used. The reversibility of the thermal transitions was checked by examining the reproducibility of the calorimetric trace in a subsequent heating of the sample immediately after cooling following the first scan. In all cases, the thermal denaturation was found to be irreversible; therefore, the thermogram corresponding to the reheating run was used as an instrumental baseline. The transitions were corrected with regard to the difference in heat capacity between the initial and final state by using a linear chemical baseline. The molar excess heat capacity curves, obtained by normalizing with the protein concentrations and the known volume of the calorimetric cell, were smoothed and plotted using the Windows-based software package (Origin). The temperature at the maximum of the excess heat capacity curve was taken as the transition temperature (*T*_*m*_). DSC measurements were carried out in buffer 20 mM HEPES, containing 100 mM NaCl, 5 mM CaCl_2_, and 5 mM MgCl_2_, pH 7.9 (20°C). The temperature coefficient of HEPES buffer is −0.014 ΔpK°C^−1^.

### 2.3. Data Analysis

The DSC data were analyzed by means of the nonlinear least-squares method using the Origin 8 software package (Origin Lab Corp.). In all fitting procedures, the correlation coefficient *r*, used as a criterion for the accuracy of fitting, was not less than 0.99. In the calculation of molar quantities, the molecular mass used for the protein was 9000 kDa.

DSC transitions were analyzed in terms of the two-state kinetic model:

(1) $$\begin{array}{c} N\overset{k}{\to }D, \end{array}$$

which is a limiting case of the Lumry–Eyring model [25]. This model considered only two significantly populated macroscopic states: the initial or native state (*N*), and the final or denatured state (*D*), the transition between which is determined by a strongly temperature-dependent first-order rate constant (*k*). The Arrhenius equation represents the temperature dependence of *k*:

(2) $$\begin{array}{c} k=\exp\left[\frac{E_{a}}{R}\left(\frac{1}{T^{*}}-\frac{1}{T}\right)\right], \end{array}$$

where *E*_*a*_ is the activation energy of the denaturation process, *R* is the gas constant, and *T*^∗^ is the temperature at which *k* is equal to 1 min^−1^.

In this case, the excess heat capacity *C*_*p*_^ex^ is given by the following equation [26]:

(3) $$\begin{array}{c} C_{p}^{\mathrm{ex}}=\frac{1}{v}\Delta H\exp\left\{\frac{E_{a}}{R}\left(\frac{1}{T^{*}}-\frac{1}{T}\right)\right\}\times \exp\left\{-\frac{1}{v}\int_{T_{0}}^{T}\exp\left[\frac{E_{a}}{R}\left(\frac{1}{T^{*}}-\frac{1}{T}\right)\text{d}T\right]\right\}, \end{array}$$

where *v* = d*T*/d*t* (K min^−1^) is a scan rate value and Δ*H* is the enthalpy difference between the denatured and native states.

## 3. Results and Discussion

### 3.1. DSC of Purified HaH Isoforms

The three Hc isoforms (*β*-HaH, *α*_*D*_-HaH, and *α*_*N*_-HaH), identified in the hemolymph of snails *H. aspersa* maxima, were isolated by ion exchange chromatography. The isoform *β*-HaH (pI 5.2) eluted as a single symmetric peak, followed by a peak containing both electrophoretically similar *α*-isoforms (pI 4.6), which eluted together as *α*_*D*+*N*_-HaH, as previously described by Raynova and coworkers [23]. At neutral pH and in the presence of Ca^2+^ and Mg^2+^, these Hc molecules occur as a didecamers of ∼450 kDa subunits.

**Figure 1:**
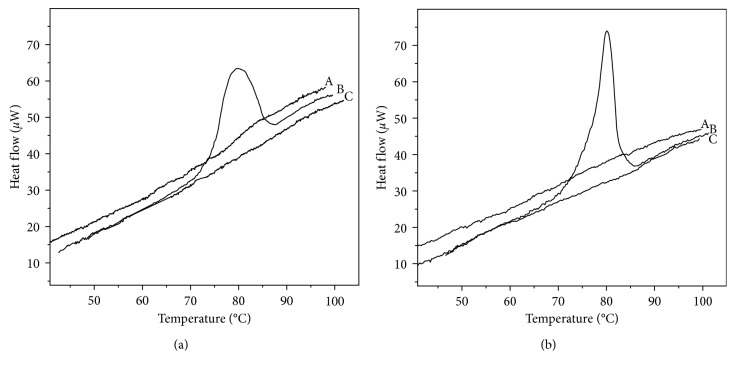
Original calorimetric recording of the apparent heat capacity of isoforms *β*-HaH (a) and *α_D+N_*-HaH (b) as a function of the temperature (line B); reheating scan (line C); and baseline obtained with buffer in both cells of the calorimeter (line A). The experimental curves shown refer to *β*-HaH (2.88 mg·ml^−1^) and *α_D+N_*-HaH (3 mg·ml^−1^) in buffer 20 mM HEPES, containing 100 mM NaCl, 5 mM CaCl_2_, and 5 mM MgCl_2_ (pH 7.06 at 80°C), at scan rate of 1.0°C min^−1^.

The thermogram of the isoform *β*-HaH in buffer 20 mM HEPES, pH 7.0, containing 100 mM NaCl, 5 mM CaCl_2_, and 5 mM MgCl_2_, at a heating rate of 1.0°C min^−1^, is shown in Figure 1(a) (line B). Heat absorption was observed between 70 and 87°C, with an apparent transition temperature (*T*_*m*_) at 79.88°C. Value of 145.0 MJ mol^−1^ was calculated for the calorimetric enthalpy (Δ*H*_cal_) by an integration of the heat capacity curve. Judging from the absence of any transitions upon rescanning the samples, the thermal denaturation of *β*-HaH was irreversible (Figure 1(a), line C).

Analysis of the heating profile of isoform *α*_*D*+*N*_-HaH, run in the same experimental conditions, revealed asymmetry of the shape, due to two overlapping transitions. In the thermogram of *α*_*D*+*N*_-HaH, one main transition with an apparent *T*_*m*_ of 80°C was observed (Figure 1(b), line B). Minor transition at ca. 76°C can also be discerned in the thermogram. The thermal denaturation of the Hc isoforms *α*_*D*+*N*_-HaH also was found to be irreversible (Figure 1(b), line C). Irreversibility of the thermal denaturation has been observed in DSC measurements of all studied to date Hcs [11–14, 27] and limits the use of a standard equilibrium thermodynamic analysis.

**Figure 2:**
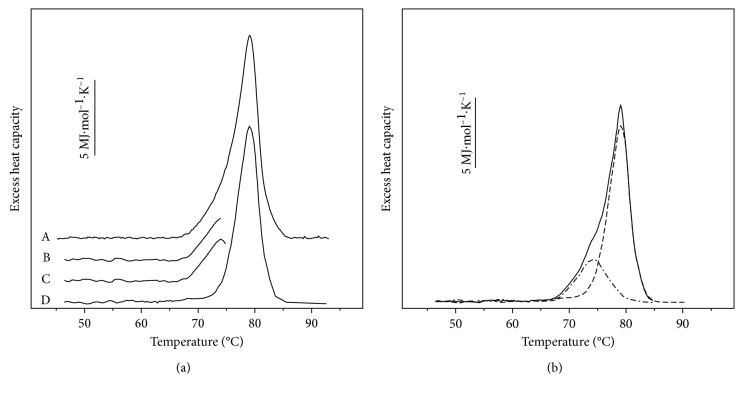
Example of the successive annealing process. (a) Complete calorimetric scan of *α*_*D*+*N*_-HaH in buffer 20 mM HEPES (pH 7.06 at 80°C), at scan rate of 0.5°C min^−1^ (line A); the first scan of a new sample with the same composition, stopped at 74.5°C (line B); result of subtracting the following (second scan) DSC line from the first one (line C); and the second calorimetric scan stopped at 90°C (line D). (b) Result of the deconvolution of the experimental contour (solid line), which provides two individual components (dash lines).

In view of the complex oligomeric Hc structure, the presence of more than one structural domains undergoing thermal unfolding more or less independently of each other is not unexpected. An analysis of DSC contours of the isoform *α*_*D*+*N*_-HaH was attempted with a successive annealing procedure, which was shown to be useful for the experimental deconvolution of completely complicated or partially irreversible thermal transitions [28, 29]. Successive annealing procedure was applied in the analysis of the thermograms of the other studied Hcs [11–13].  Figure 2 shows an example of this procedure, which provides two irreversible transitions. At first, *α*_*D*+*N*_-HaH was heated from 35°C up to 90°C to determine the number and *T*_*m*_ of expected transitions (Figure 2(a), line A). After that, a new sample of the same composition was heated slightly above the shoulder, which appeared at ∼74°C (Figure 2(a), line B) and after cooling to 35°C was reheated up to 90°C (Figure 2(a), line D). To determine the ascending part of the first transition, subtracting of the second scan from the first one was done (Figure 2(a), line C). The descending part of the curve (line C) was obtained by extrapolation (Figure 2(b), dash lines).

### 3.2. Concentration Dependence of the Temperature of Denaturation

**Figure 3:**
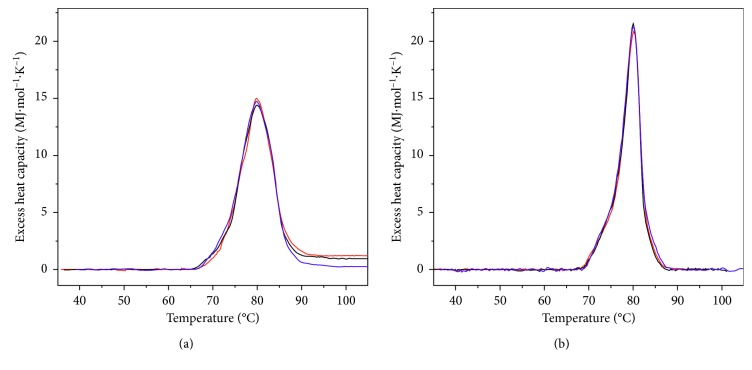
(a) *C*_*p*_ transition curve of *β*-HaH in buffer 20 mM HEPES (pH 7.06 at 80°C), recorded at scan rate of 1°C min^−1^ and different protein concentrations: 1.01 mg·ml^−1^ (blue line), 1.91 mg·ml^−1^ (red line), and 2.88 mg·ml^−1^ (black line). (b) *C*_*p*_ transition curve of *α*_*D*+*N*_-HaH in buffer 20 mM HEPES (pH 7.06 at 80°C), recorded at scan rate of 1°C min^−1^ and different protein concentrations: 2.0 mg·ml^−1^ (blue line), 3.0 mg·ml^−1^ (black line), and 4.52 mg·ml^−1^ (red line).

As is mentioned above, the quaternary structure of the Hc molecule represents oligomer of twenty subunits (didecamer). Dissociation of the Hc molecule to individual subunits may take place during the denaturation process. According to the equilibrium thermodynamics, any change in the oligomerization state of the protein during the denaturation process should produce a concentration dependence of *T*_*m*_. In order to determine whether this was occurring, DSC traces were collected at various protein concentrations, at a constant scan rate of 1°C min^−1^. DSC experiments showed that the *T*_*m*_ and calorimetric enthalpy (Δ*H*_cal_) values for the thermal unfolding of isoforms *β*-HaH and *α*_*D*+*N*_-HaH were independent of the protein concentration (Figures 3(a) and 3(b)); therefore, denatured proteins remained in the same oligomerization state as the native ones. Consistent with our previous studies, the heat absorption observed in the DSC curves (*T*_*m*_∼80°C) is connected to the melting of the compact Hc quaternary structure without simultaneous dissociation into subunits [11–13]. This indicates that the interactions between the subunits, constituting the Hc molecules, are strong enough to prevent dissociation before the rate-determining step of the process of thermal unfolding.

### 3.3. Scan Rate Dependence

**Figure 4:**
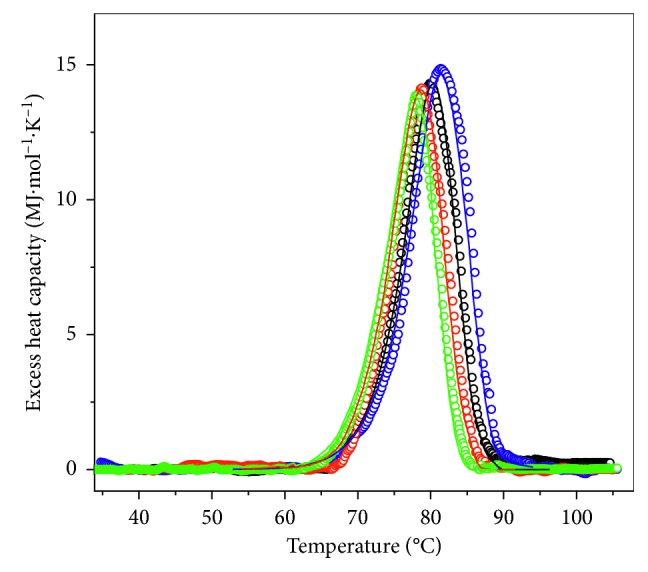
Dependence of the *C*_*p*_ transition curves of *β*-HaH in buffer 20 mM HEPES (pH 7.06 at 80°C), with a scan rate: 0.24°C min^−1^, green symbols; 0.5°C min^−1^, red symbols; 1.0°C min^−1^, black symbols; 1.5°C min^−1^, blue symbols. In all cases the protein concentration was 2.88 mg·ml^−1^. The solid lines represent the theoretical fitting curves based on (3).

In the case of irreversible thermal denaturation of proteins, the calorimetric profiles are scan rate dependent, and the process is under kinetic control.  Figure 4 shows the excess heat capacity function versus temperature profiles of the isoform *β*-HaH at four different scan rates (0.24, 0.5, 1.0, and 1.5°C min^−1^). The transition temperature of the irreversible thermal denaturation of *β*-HaH is scan rate dependent; the maximum of the DSC profiles is shifted toward lower temperatures with a reduction in scan rate (Figure 4).

**Figure 5:**
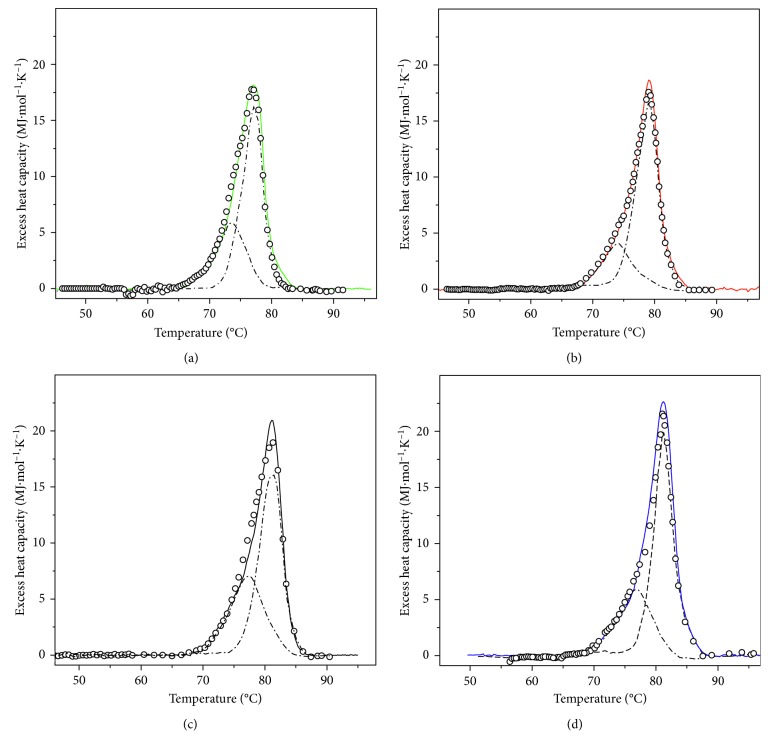
Dependence of the *C*_*p*_ transition curves of *α*_*D*+*N*_-HaH isoforms in buffer 20 mM HEPES (pH 7.06 at 80°C), with a scan rate: 0.2°C min^−1^ (a); 0.5°C min^−1^ (b); 1.0°C min^−1^ (c); 1.5°C min^−1^ (d). Continuous lines depict the experimental data; dash dot lines show the experimental individual components, result of the annealing process, and symbols represent the result of the sum of the corresponded individual components. In all cases, the protein concentration was 3 mg·ml^−1^.

The transition temperature of *α*_*D*+*N*_-HaH isoforms is also scan rate dependent, indicating that the process of thermal unfolding is kinetically controlled (Figures 5(a)–5(d)).

**Figure 6:**
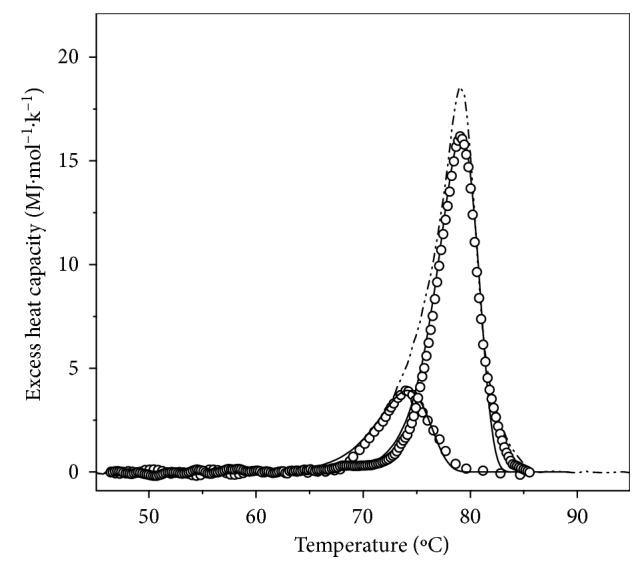
*C*_*p*_ transition curve of of *α*_*D*+*N*_-HaH isoforms in buffer 20 mM HEPES (pH 7.06 at 80°C) recorded at scan rate 0.5°C min^−1^. The solid lines represent the theoretical fitting curves based on (3), lines with symbols show the curves obtained from the deconvolution procedure, and the dash dot line represent the experimental *C*_*p*_ transition curve.

As it is evident from Figures 4 and 5, the DSC traces for both HaH isoforms were strongly dependent on the scan rate, which prompted us to analyze this nonequilibrium process based on the simplest model of irreversible thermal denaturation of proteins (1). The excess heat capacity functions were analyzed by fitting the data to the two-state irreversible model according to (3) (Figures 4 and 6). Applying this analysis on the observed transitions of the two HaH isoforms, the kinetic parameters presented in Tables 1 and 2 were obtained.

The thermal denaturation of Hcs from different species has been described by the two-state irreversible model, and the parameters of the Arrhenius equation have been calculated, allowing comparison of transitions during Hc denaturation [11–13, 26, 27]. The activation energy value of 302.0 ± 6 (kJ·mol^−1^) and 596.0 ± 10 (kJ·mol^−1^) was determined for *β*-HaH and *α*_*D*+*N*_-HaH, respectively, which is in good agreement with values reported for Hcs in related species (Table 3). The structural organization of these Hcs is similar (didecamer), so it is difficult to propose a structural basis for the difference in their thermal stability. From the other hand, a difference in the carbohydrate content of Hcs has been observed [30]. Hence, a correlation between the thermal stability of Hcs and their carbohydrate content could be expected [31]. Actually, the most thermostable *β*-Hc of *H. pomatia* contains 7% (w/w) carbohydrates [32], while carbohydrate content of 2.6% (w/w) has been determined for RtH [30]. Data about the carbohydrate content of the Hcs of gastropods *C. concholepas* and *H. aspersa* maxima, however, are still not available in the literature.

The present study confirms that the two-state irreversible model, built especially on experimental examinations on small globular proteins will hold true for larger oligomeric proteins as well [33]. Nevertheless, as Lyubarev and Kurganov noted in [34], that experimental data are satisfactorily described by the two-state model, the real mechanism of protein denaturation can be more complex. Therefore, it should be stressed that the obtained values for the activation energy of the denaturation process are apparent and characterize the initial rate-limiting step of the process.

## 4. Conclusions

The results of the present study on the isoforms of the Hc, isolated from snails *Helix aspersa* maxima, allow classifying these Hcs as thermostable proteins (*T*_*m*_∼80°C). The high degree of oligomerization of hemocyanin molecules, in general, is probably one of the reasons for their increased thermal stability. The data obtained will facilitate the further investigation of therapeutic properties and applications of these dioxygen-binding proteins.

## Figures and Tables

**Table 1:** Arrhenius equation parameters, estimated for the two-state irreversible model of the thermal denaturation of isoform *β*-HaH, at neutral pH.

Parameter	Temperature scan rate (°C min^−1^)			
	0.24	0.5	1.0	1.5
*T*_*m*_ (°C)	77.90	78.80	79.88	81.40
Δ*H*_cal_ (MJ·mol^−1^)	118.6	127.4	145.0	154.0
*E*_*a*_ (kJ·mol^−1^)	307 ± 7	316 ± 4	302 ± 6	306 ± 8
*T* ^∗^(°C)	84.6 ± 0.5	84.8 ± 0.3	85.0 ± 0.4	84.9 ± 0.3
*r*	0.992	0.993	0.996	0.994

**Table 2:** Arrhenius equation parameters, estimated for the two-state irreversible model of the thermal denaturation of isoform *α*_*D*+*N*_-HaH, at neutral pH.

Parameter	Temperature scan rate (°C min^−1^)			
	0.2	0.5	1.0	1.5
First transition				
*T*_*m*_ (°C)	73.4	74.0	76.4	77.0
Δ*H*_cal_ (MJ·mol^−1^)	31.10	34.07	39.10	41.27
*E*_*a*_ (kJ·mol^−1^)	424 ± 9	434 ± 6	418 ± 5	423 ± 7
*T* ^∗^ (°C)	80.8 ± 0.5	79.0 ± 0.3	80.0 ± 0.4	79.0 ± 0.3
*r*	0.989	0.985	0.988	0.987
Second transition^∗^				
*T*_*m*_ (°C)	77.1	79.02	80.07	81.20
Δ*H*_cal_ (MJ·mol^−1^)	69.9	79.0	79.8	81.4
*E*_*a*_ (kJ·mol^−1^)	586 ± 12	564 ± 8	596 ± 10	601 ± 14
*T* ^∗^ (°C)	83.8	83.9	83.7	83.6
*r*	0.995	0.996	0.993	0.994

^∗^Main transition.

**Table 3:** Parameters for the thermal denaturation of HaH isoforms obtained by DSC at scan rate of 1°C min^−1^. Comparison with data obtained for Hcs of *R. thomasiana* [11], *H. pomatia* [12], *C. concholepas* [13], and *H. rubra* [27].

Hemocyanin	*T*_m_^∗^ (°C)	Δ*H*cal (MJ·mol^−^^1^)	*E*_*a*_ (kJ·mol^−^^1^)
*H. aspersa β*-Hc (*β*-HaH)	79.88	145 ± 1.0	302.0 ± 6
*H. aspersa α*_*D*+*N*_-Hc (*α*_*D*+*N*_-HaH)	80.07	79.8 ± 1.0	596.0 ± 10
*R. thomasiana* Hc (RtH)	82.4	195 ± 1.0	597.0 ± 20
*H. pomatia β*-Hc (*β*-HpH)	84.0	190 ± 1.0	521.0 ± 7
*C. concholepas* Hc (CCH)	78.0	120 ± 1.0	323.0 ± 2
*H. rubra* Hc	78.9	—	535.0 ± 65

^∗^Main transition.

## Abbreviations

HaH: Hemocyanin of *Helix aspersa* maxima
DSC: Differential scanning calorimetry
Tris-HCl: Tris(hydroxymethyl)aminomethane hydrochloride
HEPES: 4-(2-Hydroxyethyl)-1-piperazineethanesulfonic acid.
